# VGG19 Network Assisted Joint Segmentation and Classification of Lung Nodules in CT Images

**DOI:** 10.3390/diagnostics11122208

**Published:** 2021-11-26

**Authors:** Muhammad Attique Khan, Venkatesan Rajinikanth, Suresh Chandra Satapathy, David Taniar, Jnyana Ranjan Mohanty, Usman Tariq, Robertas Damaševičius

**Affiliations:** 1 Department of Computer Science, HITEC University, Taxila 47080, Pakistan; attique@ciitwah.edu.pk; 2 Department of Electronics and Instrumentation Engineering, St. Joseph’s College of Engineering, Chennai, Tamilnadu 600119, India; v.rajinikanth@ieee.org; 3 School of Computer Engineering, Kalinga Institute of Industrial Technology (Deemed to Be University), Bhubaneswar, Odisha 751024, India; suresh.satapathyfcs@kiit.ac.in; 4 Faculty of Information Technology, Monash University, Clayton, VIC 3800, Australia; David.Taniar@monash.edu; 5 School of Computer Applications, Kalinga Institute of Industrial Technology (Deemed to Be University), Bhubaneswar, Odisha 751024, India; jmohantyfca@kiit.ac.in; 6 College of Computer Engineering and Sciences, Prince Sattam Bin Abdulaziz University, Al-Kharj 11942, Saudi Arabia; u.tariq@psau.edu.sa; 7 Faculty of Applied Mathematics, Silesian University of Technology, 44-100 Gliwice, Poland

**Keywords:** lung CT images, nodule detection, VGG-SegNet, pre-trained VGG19, deep learning

## Abstract

Pulmonary nodule is one of the lung diseases and its early diagnosis and treatment are essential to cure the patient. This paper introduces a deep learning framework to support the automated detection of lung nodules in computed tomography (CT) images. The proposed framework employs VGG-SegNet supported nodule mining and pre-trained DL-based classification to support automated lung nodule detection. The classification of lung CT images is implemented using the attained deep features, and then these features are serially concatenated with the handcrafted features, such as the Grey Level Co-Occurrence Matrix (GLCM), Local-Binary-Pattern (LBP) and Pyramid Histogram of Oriented Gradients (PHOG) to enhance the disease detection accuracy. The images used for experiments are collected from the LIDC-IDRI and Lung-PET-CT-Dx datasets. The experimental results attained show that the VGG19 architecture with concatenated deep and handcrafted features can achieve an accuracy of 97.83% with the SVM-RBF classifier.

## 1. Introduction

Lung cancer/nodule is one of the severe abnormalities in the lung, and a World Health Organization (WHO) report indicated that around 1.76 million deaths have occurred globally in 2018 due to lung cancer [1]. Lung cancer/nodule is due to abnormal cell growth in the lung and, in most cases, the nodule may be cancerous/non-cancerous. The Olson report [2] confirmed that lung nodules can be categorized into benign/malignant based on their dimension (5 to 30 mm fall into the benign class and >30 mm is malignant). When a lung nodule is diagnosed using the radiological approach, a continuous follow-up is recommended to check its growth rate. The follow-up procedure can continue for up to two years and, along with non-invasive radiographic imaging procedures, other invasive methodologies, such as bronchoscopy and/or tissue biopsy, can also be suggested to confirm the condition and harshness of the lung nodules in a patient [3].

Noninvasive radiological techniques are commonly adopted in initial level lung nodule detection using CT images, and, therefore, several lung nodule detection works are already proposed in the literature [4,5,6] which involve the use of traditional signal processing and texture analysis techniques combined with machine learning classification [7], deep learning models [8,9], neural networks combined with nature-inspired optimization techniques [10,11] and ensemble learning [12]. The aims of this research are to construct a Deep Learning (DL) supported scheme to segment the lung nodule segment from the CT image slice with better accuracy and classify the considered CT scan images into normal/nodule class with improved accuracy using precisely selected deep and handcrafted features. 

The recent article by Rajinikanth and Kadry [13] proposed a framework with VGG16 neural network model for the automated segmentation and classification of lung nodules from CT images. In their paper, a threshold filter technique is implemented to remove artifacts from CT images, and the artifact-eliminated images are then considered to test the proposed disease detection framework. The proposed scheme is tested using the LIDC-IDRI database [14,15,16] and the classification task implemented with the combined deep and handcrafted features helped to achieve a classification accuracy of 97.67% with a Random Forest (RF) classifier. 

In this paper, we suggest a framework to support automated segmentation and classification of lung nodules with improved accuracy. The proposed scheme includes the following stages: (i) image collection and resizing, (ii) implementing the pre-trained VGG supported segmentation; (iii) deep feature-based classification, (iv) extracting the essential handcrafted features such as Gray Level Co-occurrence Matrix (GLCM), Local Binary Pattern (LBP) and Pyramid Histogram of Oriented Gradients (PHOG), (v) implementing a serial feature concatenation to unite the deep and handcrafted features and (vi) implementing and validating the performance of the classifiers using a 10-fold cross validation. 

The images used for the experiments are collected from the LIDC-IDRI [15] and Lung-PET-CT-Dx [17] datasets. All these works are realized using the MATLAB^®^ (MathWorks, Inc., Natick, MA, USA), and the attained result is then compared and validated with the earlier results presented in the literature. 

The major contribution of the proposed work is as follows:

i. Implementation of VGG19 to construct the VGG-SegNet scheme to extract lung nodule.
ii. Deep learning feature extraction based on VGG19.
iii. Combining handcrafted features and deep features to improving nodule detection accuracy.

The proposed work is organized as follows. Section 2 presents and discusses earlier related research. Section 3 presents the implemented methodology. Section 4 shows the experimental results and discussions and, finally, the conclusions of the present research study are given in Section 5.

## 2. Related Work

Due to its impact, a significant amount of lung nodule detection from CT images is proposed using a variety of image databases, and summarizing the presented schemes will help to obtain an idea of the advantages and limitations of the existing lung nodule detection procedures. Traditional methods of machine learning (ML) and deep learning (DL) were proposed to examine lung nodules using CT image slices, and the summary of the selected DL-based lung nodule detection systems is presented in Table 1; all the considered works in this table discuss the lung nodule detection technique using a chosen methodology. Furthermore, all these works considered the LIDC-IDRI database for examination.

The summary (see Table 1) presents a few similar methods implemented using CT images of the LIDC-IDRI database, and the highest categorization accuracy achieved is 97.67% [13]. 

In addition, a detailed evaluation of various lung nodule recognition practices existing in the literature is available in the following references [25,26,27]. Some of the works discussed in Table 1 recommended the need for a competent lung nodule detection system that can support both segmentation of the nodule section and classification of lung nodules from normal (healthy) CT images. The works discussed in Table 1 implemented either a segmentation or classification technique using deep features only. Obtaining better detection accuracy is difficult with existing techniques and, hence, the combination of deep features (extracted by a trained neural network model) and handcrafted features is necessary.

In this paper, the pre-trained VGG-16 supported segmentation (VGG-SegNet) is initially executed to extract the lung nodule section from CT images, and then the CT image classification is executed using deep features as well as combined deep and handcrafted features. A detailed assessment among various two-class classifiers, such as SoftMax, Decision-Tree (DT), RF, K-Nearest Neighbor (KNN) and SVM-RBF are also presented using a 10-fold cross-validation to validate the proposed scheme.

## 3. Methodology

**Figure 1:**
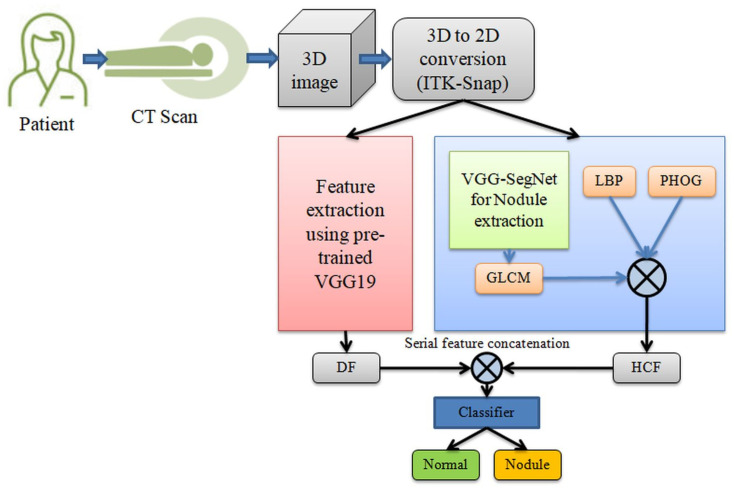
Structure of the proposed lung-nodule segmentation and classification system.

In the literature, several lung abnormality detection systems based on DL are proposed and implemented using clinical-level two-dimensional (2D) CT images as well as benchmark images. Figure 1 shows the proposed system to segment and classify the lung nodule section of the CT images. Initially, the CT images are collected from the benchmark data set and, later, the conversion from 3D to 2D is implemented using ITK-Snap [28]. The ITK-Snap converts the 3D images into 2D slices of planes, such as axial, coronal and sagittal and, in this work, only the axial plane is considered for the assessment. Finally, all test images are resized to 224 × 224 × 3 and then used for the segmentation and classification task. The resized 2D CT images are initially considered for the segmentation task; where the lung nodule segment is mined using the VGG-SegNet scheme implemented with the VGG19 architecture. Later, the essential features are extracted with GLCM, LBP and PHOG, and then these features are combined with the learned features of the pre-trained DL scheme. Finally, the serially concatenated deep features (DF) and handcrafted features (HCF) are used to train, test and confirm the classifier. Based on the attained performance values, the performance of the proposed system is validated.

### 3.1. Image Database Preparation

The CT images are collected from LIDC-IDRI [15] and Lung-PET-CT-Dx [17] databases. These data sets have the clinically collected three-dimensional (3D) lung CT images with the chosen number of slices. 

**Figure 2:**
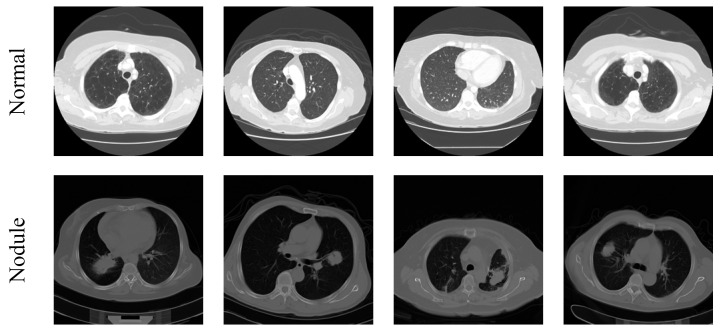
Sample test images considered in this study.

The assessment of the 3D CT images is quite complex and, hence, 3D to 2D conversion is performed to extract the initial image with a dimension of 512 × 512 × 3 pixels, and these images are then resized to 224 × 224 × 3 pixels to decrease the assessment complexity. In this work, only the axial view of 2D slices is used for the estimation and the sample test images of the considered image data set are depicted in Figure 2 and the total images for investigation are given in Table 2. 

### 3.2. Nodule Segmentation

Evaluation of the shape and dimension of the abnormality in medical images is widely preferred during the image-supported disease diagnosis and treatment implementation process [29,30]. Automated segmentation is widely used to extract the infected section from the test image and the mined fragment is further inspected to verify the disease and its severity level. In the assessment of the lung nodule with CT images, the dimension of the lung nodule plays a vital role and, therefore, the extraction of the nodule is very essential. In this work, the VGG-SegNet scheme is implemented with the VGG19 scheme to extract the CT image nodule. Information on the traditional VGG-SegNet model can be found in [29].

**Figure 3:**
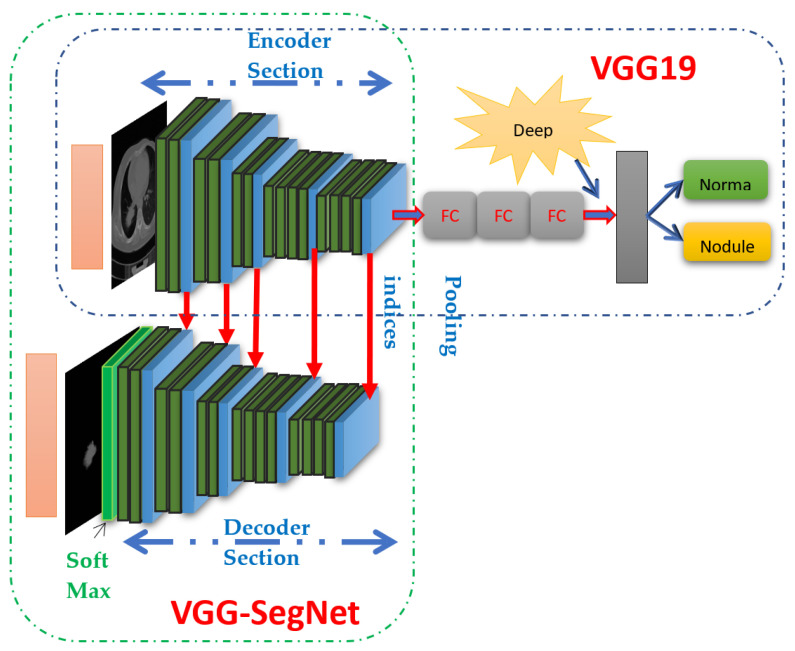
Structure of VGG19 supported segmentation (VGG-SegNet) and classification scheme.

The proposed VGG-SegNet model consists of the following specification; traditional VGG19 scheme is considered as the encoder section and its associated structure forms the decoder unit. Figure 3 illustrates the construction of the VGG19-based segmentation and classification scheme in which the traditional VGG19 scheme (first 5 layers) works as the encoder region and the inverted VGG19 with up-sampling facility is then considered as the decoder region. The pre-tuning of this scheme for the CT image is performed using the test images considered for training along with the essential image enhancement process [31]. The preliminary constraints for training the VGG-SegNet are allocated as follows: batch size is equal for encoder-decoder section, initialization uses a normal weight, learning rate is fixed as 1e-5, Linear Dropout Rate (LDR) is assigned, and Stochastic Gradient-Descent (SGD) optimization is selected. The final SoftMax layer uses a sigmoid activation function. 

### 3.3. Nodule Classification 

In the medical domain, automated disease classification plays an important role during the mass data assessment and a perfectly tuned disease classification system further reduces the diagnostic burden of physicians and acts as an assisting system during the decision-making process [32,33,34,35]. Therefore, a considerable number of disease detection systems assisted by DL are proposed and implemented in the literature [36,37,38,39,40]. Recent DL schemes implemented in the LIDC-IDRI with fused deep and HCF helped achieve a classification accuracy of >97% [13]. 

Figure 3 presents the assisted classification of using the VGG19 of lung CT images (dimension 224 × 224 × 3 pixels) using the DF using the SoftMax classifier, and then the performance of VGG19 is validated with VGG16, ResNet18, ResNet50 and AlexNet (images with dimension of 227 × 227 × 3 pixels) [41,42,43,44,45,46] and the performance is compared and validated. The performance of the implemented VGG19 is validated using DF, concatenated DF + HCF and well-established binary classifiers existing in the literature [47,48,49,50].

#### 3.3.1. Deep Features

Initially, the proposed scheme is implemented by considering the DF attained at fully connected layer 3 (FC3). After possible dropout, FC3 helps to provide a feature vector of dimension 1×1024, whose value is mathematically represented as in Equation (1).

(1) $$FV_{VGG19\;\left(1\times 1024\right)}=VGG19_{\left(1,1\right)},VGG19_{\left(1,2\right)},\ldots ,VGGgg19_{\left(1,1024\right)}$$

Other essential information on VGG19 and the related issues can be found in [41].

#### 3.3.2. Handcrafted Features

The features extracted from the test image using a chosen image processing methodology are known as Machine Learning Features (MLF) or handcrafted features (HCF). Previous research in the literature already confirmed the need for the precision of HCF to progress the categorization accuracy in a class of ML and DL-based disease detection systems [46,50,51]. In the proposed work, the essential HCF from the considered test images is extracted using well-known methods such as GLCM [13,36,42], LBP [13,46] and PHOG [48]. 

The GLCM features are commonly used due to their high performance and, in this paper, the GLCM features are extorted from the lung nodule section segmented with the VGG-SegNet. The entire feature used in this work can be found in Equation (2).

(2) $$FV1_{G\mathrm{LCM}\;\left(1\times 25\right)}=GLCM_{\left(1,1\right)},GLCM_{\left(1,2\right)},\ldots ,GLCM_{\left(1,25\right)}$$

In this work, the LBP with varied weight (weights with values; W = 1, 2, 3, and 4) is considered to mine the important features from the considered test images and the proposed LBP is already implemented in the works of Gudigar et al. [52] and Rajinikanth and Kadry [13]. The LBP features for the varied weights are depicted in Equations (3)–(6) and Equation (7) depicts the overall LBP features.

(3) $$FV_{LBP1\;\left(1\times 59\right)}=LBP1_{\left(1,1\right)},LBP1_{\left(1,2\right)},\ldots ,LBP1_{\left(1,59\right)}$$

(4) $$FV_{LBP2\;\left(1\times 59\right)}=LBP2_{\left(1,1\right)},LBP2_{\left(1,2\right)},\ldots ,LBP2_{\left(1,59\right)}$$

(5) $$FV_{LBP3\;\left(1\times 59\right)}=LBP3_{\left(1,1\right)},LBP3_{\left(1,2\right)},\ldots ,LBP3_{\left(1,59\right)}$$

(6) $$FV_{LBP4\;\left(1\times 59\right)}=LBP4_{\left(1,1\right)},LBP4_{\left(1,2\right)},\ldots ,LBP4_{\left(1,59\right)}$$

(7) $$FV2_{LBP\;\left(1\times 236\right)}=FV_{LBP1\;\left(1\times 59\right)}+FV_{LBP2\;\left(1\times 59\right)}+FV_{LBP3\;\left(1\times 59\right)}+FV_{LBP4\;\left(1\times 59\right)}$$

Along with the above said features, the PHOG features are also extracted and considered along with GLCM and LBP. The total information on the PHOG can be found in the article by Murtza et al. [48]. In this work, 255 features are extracted by assigning number of bins = 3 and levels (L) = 3. The PHOG features of the proposed work are depicted in Equation (8).

(8) $$FV3_{PHOG\;\left(1\times 255\right)}=PHOG_{\left(1,1\right)},PHOG_{\left(1,2\right)},\ldots ,PHOG_{\left(1,255\right)},$$

#### 3.3.3. Features Concatenation

In this work, a serial features concatenation is realized to unite the DF and HCF, and this technique helps to improve the feature dimension to a higher level. The serial features concatenation implemented in this work is depicted in Equation (9) and Final-Feature-Vector (FFV) is presented in Equation (10).

(9) $$Concatenated\;features=DF_{\left(1\times 1024\right)}+HCF_{\left(1\times 516\right)},$$

(10) $$FFV_{\left(1\times 1540\right)}=FV_{VGG19\;\left(1\times 1024\right)}+FV1_{GLCM\left(1\times 25\right)}+FV2_{LBP\left(1\times 236\right)}+FV3_{PHOG\left(1\times 255\right)},$$

The FFV is then used to train, test and validate the classifier considered in the proposed methodology for the automated classification of lung nodules using CT images.

#### 3.3.4. Classifier Implementation

The performance of the DL-based automated disease detection arrangement depends chiefly on the performance of the classifier implemented to categorize the considered test images based on the need. In this paper, a binary classification is initially implemented using the SoftMax classifier and, later, the well-known classifiers, such as Decision Trees (DT), RF, KNN and Support Vector Machine-Radial Basis Function (SVM-RBF) [13,53,54,55,56], are also considered to improve the classification task. In this paper, a 10-fold cross-validation process is implemented, and the finest result attained is then considered as the final classification result. The performance of the classifier is then authenticated and confirmed based on the Image Performance Values (IPV) [57,58,59].

### 3.4. Performance Computation and Validation

The overall eminence of the proposed method is validated by computing the essential IPV measures, such as True-Positive (TP), False-Negative (FN), True-Negative (TN), False-Positive (FP), Accuracy (ACC), Precision (PRE), Sensitivity (SEN), Specificity (SPE), Negative-Predicted-Value (NPV), F1-Score (F1S), Jaccard Index and Dice coeeficient, which are calculated in percentages, presented in Equations (11)–(16). The necessary information regarding these values can be found in [45,46,47].

(11) $$Accuracy=ACC=\frac{TP+TN}{TP+TN+FP+FN}\;\times \;100\%$$

(12) $$Precision=PRE=\frac{TP}{TP+FP}\;\times \;100\%$$

(13) $$Sensitivity=SEN=\frac{TP}{TP+FN}\;\times \;100\%$$

(14) $$Specificity=SPE=\frac{TN}{TN+FP}\;\times \;100\%$$

(15) $$Negative\;Predictive\;Value=NPV=\frac{TN}{TN+FN}\;\times \;100\%$$

(16) $$F1-Score=F1S=\frac{2TP}{2TP+FN+FP}\;\times \;100\%$$

(17) $$Jaccard=\frac{TP}{TP+FN+FP}\times 100\%$$

(18) $$Dice=\frac{2TP}{2TP+FN+FP}\times 100\%$$

## 4. Results and Discussions

**Figure 4:**
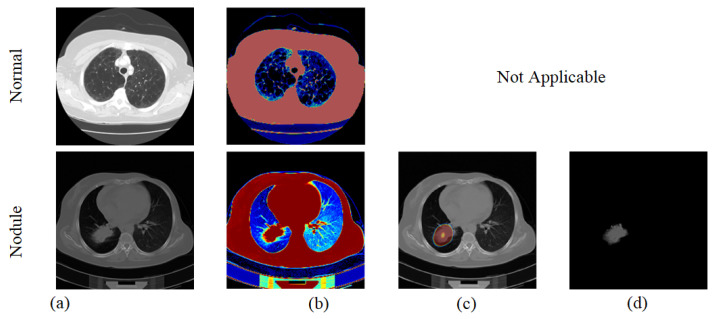
Results obtained with proposed VGG-SegNet scheme: (**a**) text image, (**b**) lung section enhanced by encoder, (**c**) localization of nodule by decoder and (**d**) extracted nodule by SoftMax unit.

This section demonstrates the results and discussions attained using a workstation with an Intel i5 2.5GHz processor, with 16GB RAM and 2GB VRAM equipped with MATLAB^®^ (version R2018a). Primarily, lung CT images are used as presented in Table 2 and then each image is resized into 224×224×3  pixels to perform the VGG19-supported segmentation and classification task. Initially, the VGG-SegNet-based lung nodule extraction process is executed on the test images considered, and the sample result obtained for the normal/nodule class image is represented in Figure 4. Figure 4 presents the experimental result of the trained VGG-SegNet with CT images. Figure 4a shows the sample images of the normal/nodule class considered for the assessment; Figure 4b depicts the outcome attained with the final layer of the encoder unit; Figure 4c,d depicts the results of the decoder and the SoftMax classifier, respectively. For the normal (healthy) class image, the decoder will not provide a positive outcome for localization and segmentation, and this section will provide the essential information only for the nodule class.

**Figure 5:**
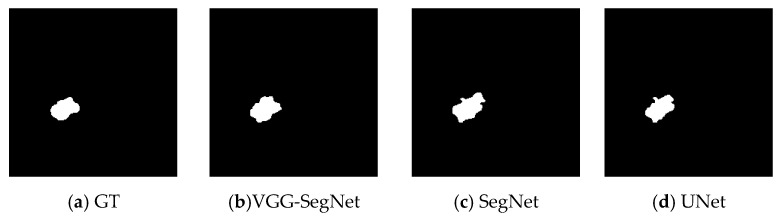
Segmentation results attained with considered CNN models.

In this paper, the extracted lung-nodule section with the proposed VGG-SegNet is compared to the ground truth (GT) image generated using ITK-Snap [28] and the essential image measures are calculated as described in previous works [4,13]. The performance of VGG-SegNet is also validated against the existing SegNet and UNet schemes in the literature [24,25,48,49]. The result achieved for the trial image is depicted in Figure 5 and Table 3, respectively. Note that the performance measures [50,51] achieved with VGG-SegNet are superior compared to other approaches.

**Figure 6:**
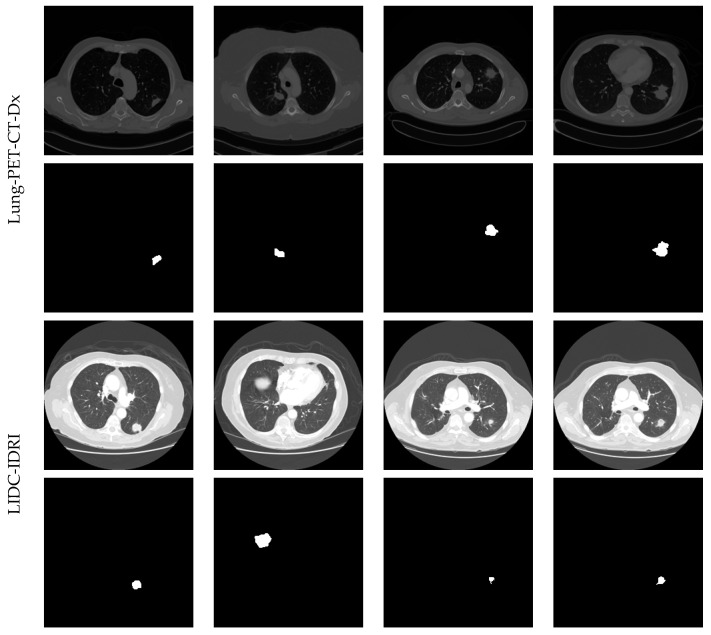
Segmentation of nodule from chosen images of Lung-PET-CT-Dx and LIDC-IDRI dataset.

The segmentation performance of the proposed scheme is then tested using the lung nodules with various dimensions, such as small, medium and large, and the attained results are depicted in Figure 6. This figure confirms that the VGG-SegNet provides a better segmentation on the medium and large nodule dimension and provides reduced segmentation accuracy on the images having lesser lung nodule due to the smaller test image dimension.

**Figure 7:**
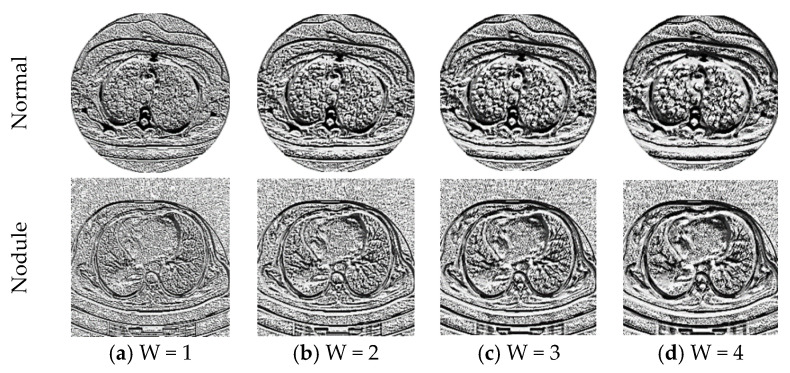
LBP patterns generated from the sample image with various LBP weights.

After collecting the essential DF with VGG19, the other HCFs, such as GLCM, LBP and PHOG are collected. The GLCM features for the normal (healthy) class image are collected from the whole CT image, and for the abnormal class image it is collected from the binary image of the extracted nodule segment. Figure 7 shows the LBP patterns generated for the normal/nodule class test images with various weight values. During LBP feature collection, each image is treated with the LBP algorithm with various weights (ie, W = 1 to 4) and the 1D features obtained from each image are combined to obtain a 1D feature vector of dimension 1×236. 

**Figure 8:**
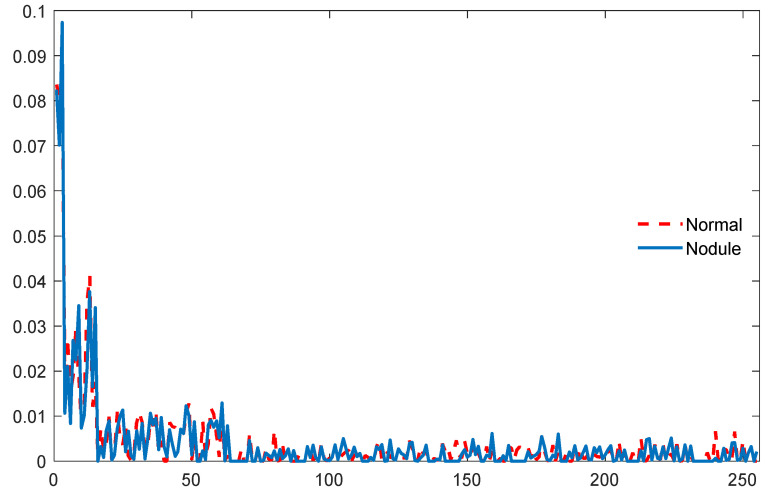
PHOG features obtained with the sample test images of Normal/Nodule class.

The PHOG features for the CT images are then extracted by assigning a bin size (L) of 3 and this process helped to obtain a 1×255 vector of features. The sample PHOG features collected for a sample CT image are seen in Figure 8. All these features (GLCM+LBP+PHOG) are then combined to form a HCF vector with a dimension of 1×516 features, following which they are then combined with the DF to improve the lung nodule detection accuracy. After collecting the essential features, the image classification task is implemented using DF and DF + HCF separately.

**Figure 9:**
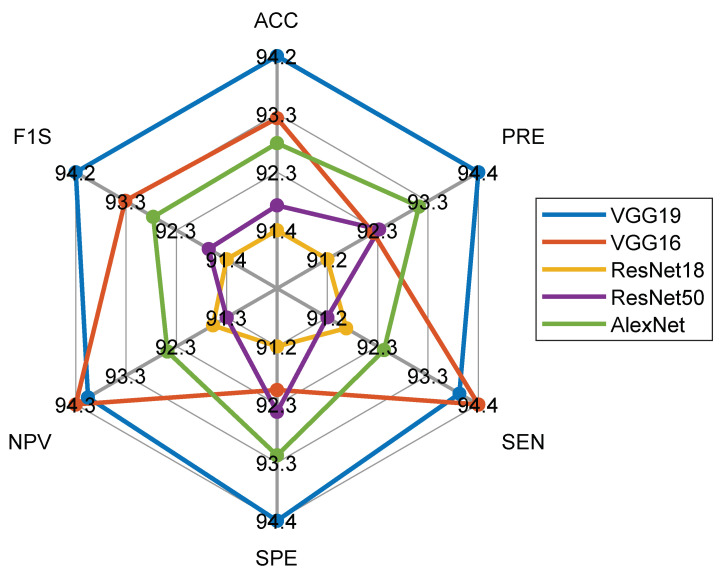
Spider plot to compare the CT image classification performance of CNN models.

Initially, the DF-based sorting is executed with the considered CNN schemes and the classification performance obtained with the SoftMax is depicted in Table 4. Figure 9 presents the spider plot for the features considered, and the result of Table 4 and the dimension of the glyph plot confirm that VGG19 helps achieve an enhanced IPV compared to other CNN schemes. VGG19 is chosen as the suitable scheme to examine the considered CT images, and then an attempt is made to enhance the performance of VGG19 using DF + HCF.

**Figure 10:**
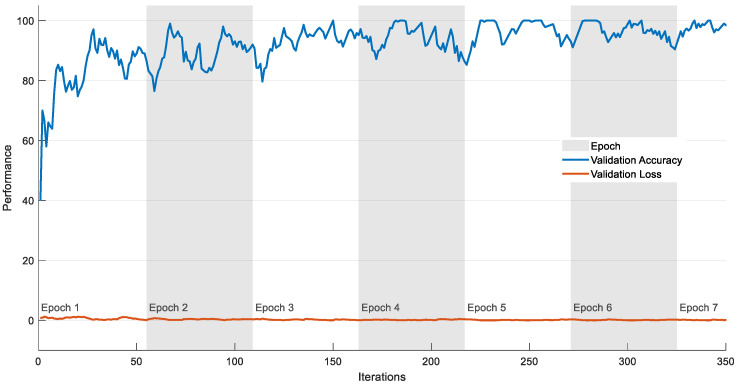
Training performance of the VGG19 with SVM-RBF for lung CT image slices.

**Figure 11:**
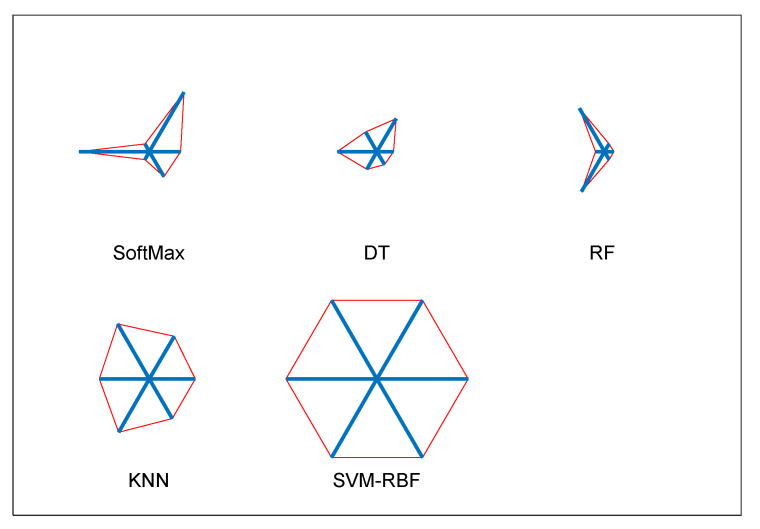
Overall performance of VGG19 with various classifiers summarized as glyph-plots.

**Figure 12:**
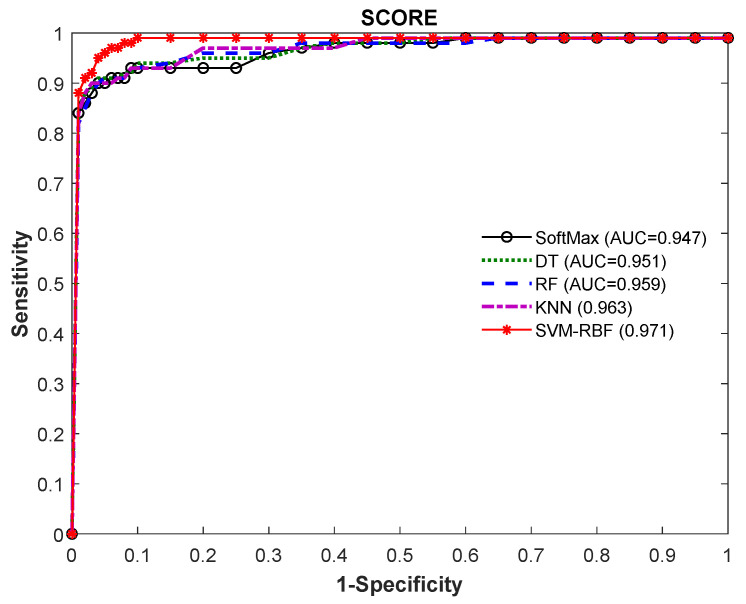
ROC curve attained for VGG19 with DF + HCF.

The experiment is then repeated using the VGG19 scheme with the DF + HCF (1 × 1540 features) using classifiers, such as SoftMax, DT, RF, KNN and SVM-RBF; the outcomes are depicted in Table 5. Figure 10 shows the performance of VGG19 with SVM-RBF, in which a 10-fold cross validation is implemented and the best result attained among the 10-fold validation is demonstrated. The result demonstrated in Table 5 confirms that the SVM-RBF classifier offers superior outcome contrast to other classifiers and a graphical illustration in Figure 11 (Glyph-Plot) also confirmed the performance of SVM-RBF. The Receiver-Operating-Characteristic curve (ROC) presented in Figure 12 also confirms the merit of proposed technique.

**Figure 13:**
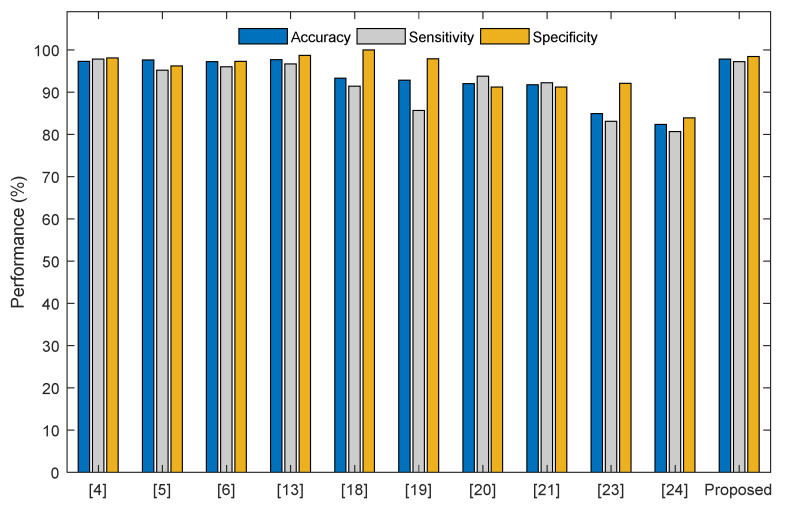
Validation of the disease detection accuracy of the proposed system with existing approaches.

The above-shown result confirms that the disease detection performance of VGG19 can be enhanced by using both the DF with the HCF. The eminence of the proposed lung nodule detection system is then compared with other methods found in the literature. Figure 13 shows the comparison of the classification precision existing in the literature and the accuracy obtained with the proposed approach (97.83%) is superior compared to other works considered for the study. This confirms the superiority of the proposed approach compared to the existing works.

The major improvement of the proposed technique compared to other works, such as Bhandary et al. [4] and Rajinikanth and Kadry [13], is as follows: this paper proposed the detection of lung nodules using CT images without removing the artifact. The number of stages in the proposed approach is lower compared to existing methods [4,10]. 

The future work includes: (i) considering other hand-made characteristics, such as HOG [48] and GLDM [43], to improve disease detection accuracy, (ii) considering the other variants of the SVM classifiers [43] to achieve better image classification accuracy and (iii) implementing a selected procedure to enhance the segmentation accuracy in lung CT having a lesser nodule size. 

## 5. Conclusions

Due to its clinical significance, several automated disease detection systems have been proposed in the literature to detect lung nodules from CT images. This paper proposes a pre-trained VGG19-based automated segmentation and classification scheme to examine lung CT images. This scheme is implemented in two stages: (i) VGG-SegNet supported extraction of lung nodules from CT images and (ii) classification of lung CT images using deep learning schemes with DF and DF + HCF. The initial part of this work implemented the VGG-SegNet architecture with VGG19-based Encoder-Decoder assembly and extracted the lung nodule section using the SoftMax classifier. Handcrafted features from the test images are extracted using GLCM (1 × 25 features), LBP with varied weights (1 × 236 features) and PHOG with an assigned bin = L = 3 (1 × 255 features), and this combination helped to obtain the chosen HCF with a dimension of 1 × 516 features. The classification task is initially implemented with the DF and SoftMax, and the result confirmed that the VGG19 provided better result compared to the VGG16, ResNet18, ResNet50 and AlexNet models. The CT image classification performance of VGG19 is once again verified using DF + HCF and the obtained result confirmed that the SVM-RBF classifier helped to obtain better classification accuracy (97.83%). 

The limitation of the proposed approach is the dimension of concatenated features (1×1540) which is rather large. In the future, a feature reduction scheme can be considered to reduce this set of features. Also, the performance of the proposed system can be improved by considering other HCFs that are known from the literature. 

## Figures and Tables

**Table 1:** Summary of existing lung nodule detection system with LIDC-IDRI database.

Reference	Lung Nodule Detection Technique	Accuracy (%)	Sensitivity (%)	Specificity (%)
Bhandary et al. [4]	A modified AlexNet with the Support Vector Machine (SVM) based binary classification helped to achieve improved result.	97.27	97.80	98.09
Choi and Choi [5]	An automated Computer-Aided-Detection scheme is proposed to examine the lung nodules using CT images.	97.60	95.20	96.20
Tran et al. [6]	A novel 15-layer DL architecture is implemented by considering the cross entropy/focal as the loss functions.	97.20	96.00	97.30
Rajinikanth and Kadry [13]	Implemented VGG16 DL scheme to segment and classify the lung nodules using deep and handcrafted features.	97.67	96.67	98.67
Kuruvilla and Gunavathi [18]	This research implemented Neural-Network (NN) supported recognition of lung nodules in CT images.	93.30	91.40	100
Nascimento et al. [19]	This work implemented a lung nodule classification based on Shannon and Simpson-Diversity Indices and SVM classifier.	92.78	85.64	97.89
Khehrah et al. [20]	Improved lung nodule detection is achieved with the help of statistical and shape features.	92.00	93.75	91.18
Wang et al. [21]	Deep NN (DNN) and 6G communication network supported lung nodule detection is proposed and implemented in this work using the CT images.	91.70	92.23	91.17
Li et al. [22]	This work implements a Convolutional-Neural-Network (CNN) supported lung nodule detection using the lung CT images.	86.40	87.10	n/a
Kaya and Can [23]	The lung nodule classification is implemented in this work and the ensemble random-forest classifier provided enhanced classification result.	84.89	83.11	92.09
Song et al. [24]	This work implemented a DNN scheme to classify the cropped lung nodule sections from the CT image slices.	82.37	80.66	83.90

**Table 2:** The lung CT images analyzed in the experiments.

Image Class	Dimension	Total Images	Training Images	Validation Images
Normal	224 × 224 × 3	1000	750	250
Nodule	224 × 224 × 3	1000	750	250

**Table 3:** Performance evaluation of CNN models on sample lung CT image. Best values are shown in bold.

Approach	Jaccard (%)	Dice (%)	ACC (%)	PRE (%)	SEN (%)	SPE (%)
VGG-SegNet	**82.6464**	**90.4988**	**99.6811**	**98.4496**	83.7363	**99.9756**
SegNet	73.1898	84.5198	99.4539	96.6408	75.1004	99.9471
UNet	79.2308	88.4120	99.6233	93.1525	**84.1307**	99.8925

**Table 4:** Classification performance attained with pre-trained DL scheme with DF and SoftMax classifier. Here TP—true positives, FN—false negatives, TN—true negatives, FP—false positives, ACC—accuracy, PRE—precision, SEN—sensitivity, SPE—specificity, NPV—negative predictive value and F1S—F1-score.

DL Scheme (Image Size)	TP	FN	TN	FP	ACC (%)	PRE (%)	SEN (%)	SPE (%)	NPV (%)	F1S (%)
VGG19 (224 × 224 × 3)	235	15	236	14	94.20	94.38	94.00	94.40	94.02	94.19
VGG16 (224 × 224 × 3)	236	14	230	20	93.20	92.19	94.40	92.00	94.26	93.28
ResNet18 (224 × 224 × 3)	229	21	228	22	91.40	91.23	91.60	91.20	91.57	91.42
ResNet50 (224 × 224 × 3)	228	22	231	19	91.80	92.31	91.20	92.40	91.30	91.75
Ale × Net (2274 × 227 × 3)	231	19	233	17	92.80	93.14	92.40	93.20	92.46	92.77

**Table 5:** Disease detection performance of VGG19 with DF + HCF with different classifiers. Best values are shown in bold.

Classifier	TP	FN	TN	FP	ACC (%)	PRE (%)	SEN (%)	SPE (%)	NPV (%)	F1S (%)
SoftMax	237	13	244	6	96.20	97.53	94.80	97.60	94.94	96.14
DT	238	12	241	9	95.80	96.36	95.20	96.40	95.25	95.77
RF	240	10	238	12	95.60	95.24	96.00	95.20	95.97	95.62
KNN	241	9	242	8	96.60	96.79	96.40	96.80	96.41	96.59
SVM-RBF	**243**	**7**	**246**	**4**	**97.83**	**98.38**	**97.20**	**98.40**	**97.23**	**97.79**

## Data Availability

The image dataset of this study can be accessed from; https://wiki.cancerimagingarchive.net/display/Public/LIDC-IDRI.
